# Three-dimensional dental arch changes of patients submitted to orthodontic-surgical treatment for correction of Class II malocclusion

**DOI:** 10.1590/2176-9451.19.4.071-079.oar

**Published:** 2014

**Authors:** Adriano Porto Peixoto, Ary dos Santos Pinto, Daniela Gamba Garib, João Roberto Gonçalves

**Affiliations:** 1 PhD resident in Oral and Maxillofacial Surgery, School of Dentistry – State University of São Paulo/Araraquara.; 2 Full Professor, Department of Orthodontics, School of Dentistry - State University of São Paulo/Araraquara.; 3 Full Professor, Department of Orthodontics. Hospital of Rehabilitation of Craniofacial Anomalies, School of Dentistry — University of São Paulo/Bauru.; 4 Assistant Professor, Department of Orthodontics, School of Dentistry-State University of São Paulo/Araraquara.

**Keywords:** Orthodontics, Orthognathic surgery, Malocclusion, Dental models

## Abstract

**Introduction:**

This study assessed the three-dimensional changes in the dental arch of patients submitted to orthodontic-surgical treatment for correction of Class II malocclusions at three different periods.

**Methods:**

Landmarks previously identified on upper and lower dental casts were digitized on a three-dimensional digitizer MicroScribe-3DX and stored in Excel worksheets in order to assess the width, length and depth of patient's dental arches.

**Results:**

During orthodontic preparation, the maxillary and mandibular transverse dimensions measured at the premolar regions were increased and maintained throughout the follow-up period. Intercanine width was increased only in the upper arch during orthodontic preparation. Maxillary arch length was reduced during orthodontic finalization, only. Upper and lower arch depths were stable in the study periods. Differences between centroid and gingival changes suggested that upper and lower arch premolars buccaly proclined during the pre-surgical period.

**Conclusions:**

Maxillary and mandibular dental arches presented transverse expansion at premolar regions during preoperative orthodontic preparation, with a tendency towards buccal tipping. The transverse dimensions were not altered after surgery. No sagittal or vertical changes were observed during the follow-up periods.

## Abstract (pt)

**Introdução:**

esse estudo avaliou as alterações tridimensionais ocorridas na morfologia das arcadas dentárias de pacientes submetidos ao tratamento ortodôntico-cirúrgico para a correção da má oclusão de Classe II, em três períodos distintos: (T_1_) modelos iniciais, (T_2_) modelos pré-cirúrgicos imediatos (de 1 a 15 dias antes da cirurgia) e (T_3_) modelos pós-cirúrgicos (mínimo de 6 meses após a remoção do aparelho ortodôntico).

**Métodos:**

pontos previamente demarcados em cada modelo foram digitalizados por meio do digitalizador tridimensional MicroScribe-3DX, cujas coordenadas, armazenadas em planilhas do programa Excel, deram origem a valores que possibilitaram a avaliação da largura, comprimento e profundidade das arcadas dentárias.

**Resultados:**

durante o preparo ortodôntico, houve aumentos nas distâncias transversais superiores e inferiores medidas na região de pré-molares que se mantiveram no período total de acompanhamento. Apenas a distância intercaninos superior apresentou alterações de aumento durante o preparo ortodôntico, assim como a largura da arcada superior, que diminuiu durante a fase de finalização. A profundidade de ambas as arcadas manteve-se estável nas fases avaliadas. Diferenças entre as mudanças dos pontos centroide e gengival sugerem que os pré-molares superiores e inferiores inclinaram para vestibular durante o preparo ortodôntico pré-cirúrgico.

**Conclusões:**

conclui-se que as arcadas dentárias superiores e inferiores sofreram expansão transversal na região de pré-molares durante o preparo ortodôntico pré-cirúrgico, com tendência à inclinação vestibular de todos os dentes posteriores. Após a cirurgia, as dimensões transversais não foram afetadas. Não foram observadas alterações sagitais ou verticais durante os períodos de observação.

## INTRODUCTION

An increasing number of adult patients seek orthodontic treatment not only for esthetic reasons, but also due to recent improvements in socioeconomic conditions. This new perspective raised the need to investigate skeletal and dental changes in soft tissue morphology occurring in adult individuals, considering the increasing search for orthodontic and orthognathic treatment.^1^

Knowledge on these changes in adulthood may help to determine if changes observed after orthodontic treatment occur primarily due to orthodontic relapse or are part of the natural process of development and maturation.^2^

Harris^3^ highlighted that changes in shape and size of the craniofacial dentoskeletal complex do not cease with biological maturity. Adulthood does not necessarily correspond to a period of absence of growth; even though change rates are lower and growth directions may be different than observed in children and adolescents. Therefore, changes occur, especially in the long term.

Long-term studies assessed the postoperative changes of orthodontically treated cases. In general, there is a tendency towards continuous reduction in the width and length of dental arches, with increase in crowding, overbite and overjet. The greatest problem has been the inability to determine whether these changes occur primarily as a result of orthodontic treatment, or if they are part of the natural maturation process.^4^

The stability of surgical changes in transverse dimensions has not been extensively assessed. Few specific studies^5,6^ investigated the stability of dental arches. Moreover, these few studies have important limitations because they do not describe the surgical technique employed and do not differentiate orthodontic relapse (dental) from surgical relapse (skeletal). An investigation with good methodology was conducted by Martin^7^ to assess the three-dimensional changes occurring in the maxillary dental arch of patients submitted to segmented osteotomy in a long-term follow-up.

In this context, this study aims at assessing the three-dimensional changes occurring in the dental arch morphology of patients submitted to orthognathic surgery for correction of skeletal Class II malocclusions.

## MATERIAL AND METHODS

This retrospective study was conducted with 15 patients (10 females and 5 males) with skeletal Class II division 1 malocclusion (Table 1) whose files were obtained from the Center for Research and Treatment of Orofacial Deformities (CEDEFACE, Araraquara, São Paulo, Brazil) and a private maxillofacial surgery practice. Dental casts were obtained at three periods: (T_1_) initial, (T_2_) immediate preoperative (1 to 15 days before surgery) and (T_3_) postoperative (minimum 6 months after the orthodontic appliance was removed). The following inclusion criteria were applied: 1) presence of all permanent teeth erupted and present in the dental arches at least from the maxillary right first molar to the maxillary left first molar; 2) dental casts with good conditions for analysis; 3) absence of anomalies of shape, incisal or occlusal abrasion, coronal fracture, caries or restorations requiring reconstruction during the study period; 4) absence of other craniofacial deformities, syndromes or cleft lip and palate; 5) preoperative and postoperative orthodontic treatment conducted without mechanical expansion or tooth extraction; 6) patients submitted to a single orthognathic surgery on one or both jaws; 7) patients older than 18 years old at surgery.

**Table 1:** Descriptive sample data.

Variable	Female (n = 10)	Male (n = 5)	Total (n = 15)
	Mean ± SD	Mean ± SD	Mean ± SD
Age / onset	27y 5m ± 8y 11m	20y 7m ± 3y 7m	25y 2m ± 8y 1m
Age / surgery	30y 0m ± 8y 11m	25y 7m ± 3y 9m	28y 6m ± 7y 9m
TOrtho	2y 7m ± 1y 5m	4y 12m ± 1y 9m	3y 5m ± 1y 10m
TSurg	1y 1m ± 0y 8m	2y 0m ± 0y 9m	1y 5m ± 0y 9m
Ttotal	3y 8m ± 2y 1m	6y 12m ± 2y 6m	4y 10m ± 2y 7m

Patients comprising the sample were operated by means of the following surgical techniques: single-piece Le Fort I osteotomy combined with bilateral sagittal split mandibular osteotomy, or isolated bilateral sagittal split mandibular osteotomy.

The method employed in this retrospective study was similar to that described by Martin^7^ who used a three-dimensional digitizer MicroScribe-3DX (3D Digitizer - The Imaging Technology Group, Illinois, USA) for digitization of predetermined landmarks on the dental casts, following the method described by Moyers et al.^8^ The software was developed for digitization and automatic storage of captured coordinates by registry in X, Y and Z coordinates on the Excel software (Microsoft Windows - Excel 12.0 - Office 2007).

**Figure 1:**
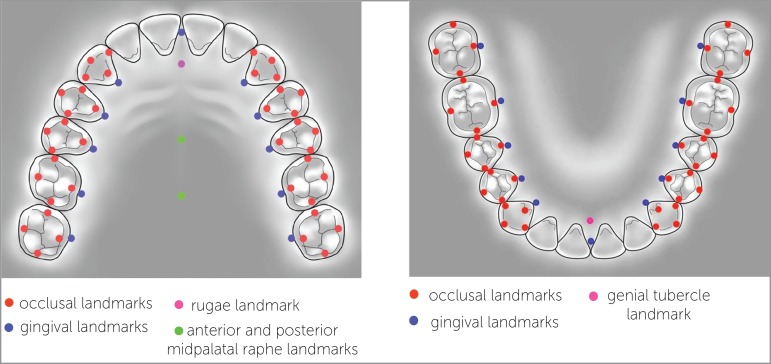
Landmarks on the maxillary dental cast.

A total of 54 landmarks were identified on the maxillary arch and 52 on the mandibular arch (Fig 1) from second molar (when present) to te canines at both sides including: mid-distal, mid-buccal, mid-mesial, mid-palatal, and gingival, each individually identified for each tooth. A gingival landmark was also identified between central incisors, the most anterior landmark in the dental arches (midline landmark = MP, Fig 1). Additional landmarks were also identified on the maxillary dental arch, namely: the rugae landmark (most posterior landmark on the incisive papilla), two landmarks on the palate (midpalatal raphe), being the first (anterior midpalatal raphe = AMR) between the first and second premolars and the second (posterior midpalatal raphe = PMR) at the mid-region of the first molar, following the position of the gingival landmark. On the mandibular dental arch, a mid-point was identified between the genial tubercles (a small rounded elevation on the lingual surface of the mandible on either side of the midline near the inferior border of the body of the mandible). The gingival landmark was identified on the most convex point of the gingival margin on the lingual aspect of each tooth. This process was repeated for each dental cast at different periods (T_1_, T_2_ and T_3_).

Dental casts were measured by a single examiner who was previously calibrated. Method error was assessed by intraclass correlation coefficient (ICC). For that purpose, all 15 triads of dental casts were digitized at two different periods, with a one-week interval.

At T_2_, for digitization of gingival landmarks obtained at the region of first and second molars (when present), the thickness of the band was subtracted, because this situation differs from T_1_ and T_3_, when the patients were not wearing any fixed appliances. This was performed considering the mean thickness (0.20 mm) of bands of the main brands commercially available in Brazil (Abzil, Morelli).

All landmarks were digitized on each dental cast (T_1_, T_2_ and T_3_) and coordinates were stored in Excel worksheets specifically developed for that purpose.

**Figure 2:**
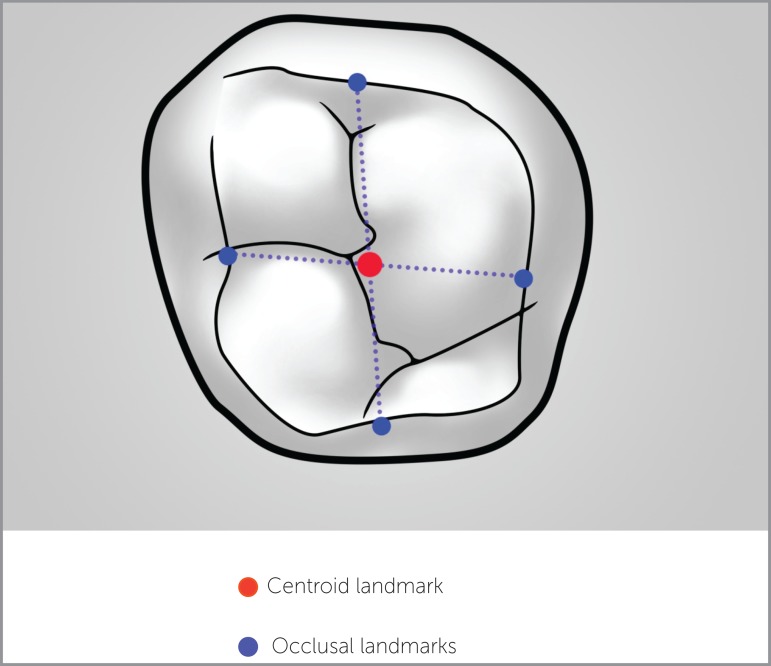
Identification of the centroid landmark.

After identifying and recording all landmarks, the centroid landmarks were calculated for each tooth (Fig 2) using the values obtained on the X, Y and Z axis between the mid-distal and buccal-palatal landmarks, as described by Moyers et al.^8^ As a result, the process obtained measurements that are relatively independent from cusp wear and are sensitive to crown translation and tooth inclination.^5^

Transverse dimensions were calculated between canines (W3-3), first premolars (W4-4), seconds premolars (W5-5), first molars (W6-6) and second molars (W7-7) (when present) at both sides, both on the centroid landmarks (C) of crowns and on gingival margins (G) of teeth.

**Figure 3:**
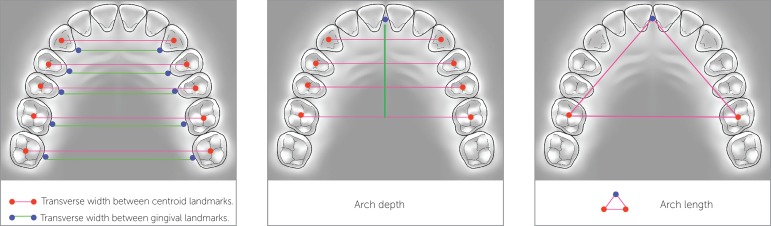
Width (**A**), depth (**B**) and length (**C**) measurements on maxillary dental cast.

Arch depths were measured from the gingival landmark between central incisors perpendicular to a line connecting the centroids of canines (D33-RUGAE), premolars (D44-AMR) and first molars (D66-PMR) for the maxillary dental arch, and D66-MP for the mandibular dental arch. Values were calculated on a software developed on the Excel system which subtracted the distance between landmarks identified on the palate in relation to a constructed transverse line. Arch length (L66-MP) was measured from the gingival landmark between central incisors to the centroid landmark of first molar on both sides (Fig 3).

Differences in measurements between the study periods determined the three-dimensional changes occurring in the dental arches during preoperative orthodontic treatment (T_2_-T_1_) and after treatment completion (T_3_-T_2_). The total differences in treatment were also calculated, including the postoperative period (T_3_-T_1_).

Data were processed and analyzed on the statistical software SPSS version 15.0 (SPSS Inc, Chicago, Il, USA) for Microsoft Windows. The hypothesis of equality of means at the three periods for each variable was analyzed using the procedure general linear model - repeated measure.

## RESULTS

The hypothesis was rejected when the p-value associated with the Hotteling-Lawley Trace was lower than 0.05. The means of variables for which this hypothesis was rejected when compared two by two by Bonferroni test for multiple comparisons of means. Test power is also presented for these variables. The correspondence of tooth movement (centroid) and skeletal movement (gingival) was compared by Student t-test for paired samples and Pearson correlation coefficient. The sample comprised 10 females and 5 males with mean ages of 27.5 and 20.7 years, respectively, at treatment onset.

### Mandibular arch

The transverse dimension between the centroid landmarks of second molars (W7-7) reduced in 0.58 mm after surgery (T_3_-T_2_). Differences among the measured widths in the centroid landmarks and measured widths in the gingival landmarks (W7-7C x W7-7G), indicative of buccal lingual inclinations, showed an increase of 0.65 mm during the pre-surgical phase (T_2_-T_1_) and a reduction of 0.54 mm in the post-surgical period (T_3_-T_2_), returning to the initial dimensions (T_3_-T_1_).

The difference in width between centroid and gingival landmarks (W6-6C x W6-6G) increased in 0.89 mm during the pre-surgical period (T_2_-T_1_) and reduced in 1.2 mm after surgery (T_3_-T_2_), returning to the initial values at the final evaluation, T_3_-T_1_ (Table 3).

**Table 3:** Comparison of mean changes between centroid and gingival landmarks. Means and standard deviation of differences between changes, t—test for the hypothesis that changes are equal and correlation coefficient between changes. Mandibular arch.

Variables	Differences between changes				T-test	r
	Study period	Mean ± SD	t	DF	p	
W7-7C x W7-7G	T_2_ - T_1_	**0.65 ±** 0.72	3.10	11	**0.010**	0.79**
	T_3_ - T_2_	**-0.54 ±** 0.55	-3.43	11	**0.006**	0.76**
	T_3_ - T_1_	0.10 ± 0.82	0.44	11	0.671	0.72**
W6-6C x W6-6G	T_2_ - T_1_	**0.89 ±** 0.71	4.69	13	0.000	0.87***
	T_3_ - T_2_	**-1.20 ±** 0.98	-4.57	13	0.001	0.65*
	T_3_ - T_1_	-0.31 ± 0.95	-1.23	13	0.241	0.80***
W5-5C x W5-5G	T_2_ - T_1_	**0.40 ±** 0.45	3.44	14	**0.004**	0.98***
	T_3_ - T_2_	-0.18 ± 0.55	-1.24	14	0.235	0.93***
	T_3_ - T_1_	0.22 ± 0.59	1.45	14	0.168	0.94***
W4-4C x W4-4G	T_2_ - T_1_	**0.59 ±** 0.64	3.58	14	**0.003**	0.97***
	T_3_ - T_2_	-0.11 ± 0.57	-0.78	14	0.451	0.83***
	T_3_ - T_1_	**0.48 ±** 0.80	2.32	14	**0.036**	0.93***
W3-3C x W3-3G	T_2_ - T_1_	-0.34 ± 0.93	-1.43	14	0.175	0.87***
	T_3_ - T_2_	-0.04 ± 0.44	-0.36	14	0.725	0.87***
	T_3_ - T_1_	-0.38 ± 0.91	-1.64	14	0.123	0.88***

*, **, *** Statistically significant correlation coefficient with significance level set at 0.05; 0.01 and 0.001, respectively.

The width between second premolars (W5-5) increased during orthodontic preparation (centroid: +1.69; gingival: +1.29), and remained stable from T_2_ to T_3_ (Table 2). The differences between centroid and gingival landmarks (W5-5C x W5-5G) increased in 0.4 mm during the pre-surgical period (T_2_-T_1_) (Table 3).

**Table 2:** Sample size (n), mean, standard deviation of changes between the two study periods, results of tests of equality of repeated measures means (means equals to zero) and multiple comparison of means. Mandibular arch.

Variable	n	T_2_-T_1_	T_3_-T_2_	T_3_-T_1_	Hotteling-Lawley Trace			Test power
		Mean ± SD	Mean ± SD	Mean ± SD	F	DF	p-value	
W7-7C	12	0.78 ± 1.15	-0.58* ± 0.69	0.20 ± 1.16	5.05	2; 10	0.030	0.682
W7-7G	12	0.14 ± 1.09	-0.04 ± 0.84	0.10 ± 0.92	0.09	2; 10	0.911	
W6-6C	14	0.66 ± 1.39	-0.70 ± 1.28	-0.03 ± 1.46	2.41	2; 12	0.132	
W6-6G	14	-0.22 ± 1.01	0.50 ± 0.93	0.28 ± 0.79	2.13	2; 12	0.162	
W5-5C	15	1.69** ± 1.86	-0.47 ± 1.32	1.22* ± 1.69	5.84	2; 13	0.016	0.780
W5-5G	15	1.29* ± 1.59	-0.29 ± 0.96	1.00 ± 1.43	4.64	2; 13	0.030	0.676
W4-4C	15	2.41** ± 2.36	-0.37 ± 1.01	2.04** ± 2.08	7.38	2; 13	0.007	0.871
W4-4G	15	1.81* ± 2.10	-0.26 ± 0.89	1.56* ± 1.73	5.71	2; 13	0.017	0.770
W3-3C	15	0.23 ± 1.83	-0.15 ± 0.64	0.08 ± 1.94	0.51	2; 13	0.612	
W3-3G	15	0.57 ± 1.40	-0.11 ± 0.86	0.46 ± 1.69	1.34	2; 13	0.295	
L66-MP	14	0.85 ± 1.28	-0.34 ± 0.66	0.51 ± 1.14	3.22	2; 12	0.076	
D66-MP	14	0.67* ± 0.81	-0.16 ± 0.75	0.51 ± 1.14	4.73	2; 12	0.031	0.675

*, **, *** account for means of changes statistically different from zero with significance level set at 0.05; 0.01 and 0.001, respectively, detected by Bonferroni's test for multiple comparison of repeated measurements means.

The width between first premolars (W4-4 C and G) showed similar results, as observed for second premolars at both study periods: T_2_-T_1_ centroid: +2.41; gingival: +1.81, T_3_-T_2_: stable. The differences between centroid and gingival landmarks showed great values to centroid landmarks (0.59 mm) during the pre-surgical period (T_2_-T_1_) and remained stable after surgery, T_3_-T_2_ (Table 3).

Dental arch length (L66-MP) and depth (D66- MP) were stable during the study periods, except for the depth assessed during orthodontic preparation which increased in 0.67 mm (Table 2).

### Maxillary arch

W6-6G remained stable during orthodontic preparation and increased in 0.86 mm after surgery (T_3_-T_2_). Comparison between T_3_-T_1_ revealed an increase of 1.11 mm in W6-6C. The difference between centroid and gingival landmarks (W6-6C x W6-6G) increased in 1.18 mm during the pre-surgical period (T_2_-T_1_) (Table 5).

**Table 5:** Means and standard deviation of differences between centroid and gingival landmarks, means and standard deviation of differences of changes, t-test for the hypothesis that changes are equal and correlation coefficient between changes. Maxillary arch.

Variables	Difference between changes		T-test			r
	Study period	Mean ± SD	t	DF	p	
W7-7C x W7-7G	T_2_ - T_1_	0.41 ± 1.01	1.46	12	0.169	0.81***
	T_3_ - T_2_	-0.11 ± 0.58	-0.70	12	0.496	0.98***
	T_3_ - T_1_	0.30 ± 0.89	1.20	12	0.252	0.86***
W6-6C x W6-6G	T_2_ - T_1_	1.18 ± 0.77	5.93	14	0.000	0.92***
	T_3_ - T_2_	-0.27 ± 0.72	-1.45	14	0.169	0.84***
	T_3_ - T_1_	0.91 ± 0.81	4.35	14	0.001	0.90***
W5-5C x W5-5G	T_2_ - T_1_	1.54 ± 1.36	4.38	14	0.001	0.81***
	T_3_ - T_2_	-0.13 ± 0.71	-0.69	14	0.501	0.76**
	T_3_ - T_1_	1.42 ± 1.25	4.38	14	0.001	0.74**
W4-4C x W4-4G	T_2_ - T_1_	1.04 ± 1.20	3.36	14	0.005	0.91***
	T_3_ - T_2_	0.04 ± 0.76	0.18	14	0.857	0.80***
	T_3_ - T_1_	1.08 ± 1.00	4.18	14	0.001	0.93***
W3-3C x W3-3G	T_2_ - T_1_	0.49 ± 0.97	1.94	14	0.072	0.91***
	T_3_ - T_2_	-0.23 ± 0.34	-2.60	14	0.021	0.95***
	T_3_ - T_1_	0.25 ± 1.01	0.98	14	0.343	0.88***

*, **, *** Statistically significant correlation coefficient with significance level set at 0.05; 0.01 and 0.001, respectively.

W5-5G (+0.96) and C (+2.51) distances increased during orthodontic preparation and remained stable from T_2_ to T_3_ (Table 4). Differences between centroid and gingival landmarks (W5-5C x W5-5G) increased in 1.54 mm during pre-surgical orthodontic preparation (T_2_-T_1_) (Table 5).

**Table 4:** Sample size (n), mean, standard deviation of changes between the two study periods, results of tests of equality of repeated measures means (means equals to zero) and multiple comparison of means. Maxillary arch.

Variable		T_2_-T_1_	T_3_-T_2_	T_3_-T_1_	Hotteling-Lawley Trace			Test power
	n	Mean ± SD	Mean ± SD	Mean ± SD	F	DF	p-value	
W7-7C	13	0.26 ± 1.60	0.20 ± 2.34	0.46 ± 1.72	0.59	2; 11	0.573	
W7-7G	13	-0.15 ± 1.65	0.31 ± 1.94	0.16 ± 1.40	0.16	2; 11	0.856	
W6-6C	15	0.52 ± 1.90	0.59 ± 1.32	1.11* 1.46	5.12	2; 13	0.023	0.721
W6-6G	15	-0.66 ± 1.56	0.86* 1.18	0.20 ± 0.82	4.74	2; 13	0.029	0.686
W5-5C	15	2.51** ± 2.19	0.11 ± 1.02	2.61*** ± 1.86	14.10	2; 13	0.001	0.991
W5-5G	15	0.96* 1.34	0.23 ± 1.02	1.20** ± 1.32	5.84	2; 13	0.016	0.780
W4-4C	15	3.29*** ± 2.50	-0.14 ± 1.29	3.15*** ± 2.28	13.75	2; 13	0.001	0.990
W4-4G	15	2.25*** ± 1.67	-0.18 ± 1.04	2.07*** ± 1.59	13.65	2; 13	0.001	0.990
W3-3C	15	1.72* 2.11	-0.52 ± 1.11	1.19 ± 2.04	4.95	2; 13	0.025	0.706
W3-3G	15	1.23* 1.48	-0.29 ± 1.02	0.94 ± 1.53	4.85	2; 13	0.027	0.697
L66-MP	15	-0.07 ± 2.25	-0.74** ± 0.80	-0.81 ± 2.14	6.69	2; 13	0.010	0.836
D33-RUGAE	15	-0.09 ± 0.83	-0.01 ± 0.39	-0.10 ± 0.81	0.11	2; 13	0.845	
D44-AMR	15	-0.12 ± 1.36	-0.54 ± 1.43	-0.66 ± 1.26	2.03	2; 13	0.171	
D66-PMR	15	-0.20 ± 0.65	0.26 ± 0.59	0.06 ± 0.72	1.53	2; 13	0.253	

*, **, *** Account for means of changes statistically different from zero with significance level set at 0.05; 0.01 and 0.001, respectively, detected by Bonferroni's test for multiple comparison of repeated measurements means.

The same behavior was observed for W4-4 C (+3.29 mm) and W4-4 G (+2.25 mm) distances that increased during the pre-surgical period. Differences between centroid and gingival landmarks (W4-4C x W4-4G) increased in 1.04 mm during the pre-surgical period and remained stable after surgery.

At the region 3-3, there was an increase of 1.72 mm between centroids and 1.23 mm in the gingival landmark between T_1_ and T_2_. Differences between centroid and gingival landmarks (W3-3C x W3-3G) decreased in 0.23 mm in the post-surgical period (Table 5).

Arch length (L66-MP) remained stable during orthodontic preparation (T_2_-T_1_) and reduced in -0.74 mm from T_2_ to T_3_. Arch depth remained stable at all study periods (Table 4).

## DISCUSSION

This study analyzed the three-dimensional changes occurring in the maxillary and mandibular dental arches of patients submitted to orthognathic surgery at two different periods: during preoperative orthodontic preparation and in the postoperative follow-up. The postoperative period included patients monitored for at least 6 months after the orthodontic appliance was removed with a mean period of postoperative evaluation of 1.1 years for females and 2 years for males (Table 1). Patients used retainers after removal of fixed appliances for an average period of 6 months. This period was adequate for assessing the most critical period of stability. No long-term evaluations were included to reduce the chance of influence from slight dental arches changes after growth completion, as described in the literature,^2,3,9,10,11^ since these changes were observed in 10-year to 34-year longitudinal studies.

Comparison with an untreated group would be valuable, since dimensional changes in the dental arches continue to occur even after post-pubertal growth.^2,3,11,12,13^ Description of changes that naturally occur in untreated individuals may be taken as gold standard to assess the changes caused by orthodontic treatment.^13^ The difficulty to achieve a paired group in terms of age, sex and type of malocclusion, as well as the ethical aspect concerning the impossibility to offer treatment during the study period (58 months) led to the decision to include a single group in this study.

Dimensional changes in the dental arches of untreated individuals are known, yet some divergences still persist among authors. Nevertheless, the described changes are of small magnitude (smaller than 1 mm) for a study period of 10 to 34 years, with a tendency towards narrowing and shortening of maxillary and mandibular dental arches over time. Bondevik^14^ reported different results, with changes slightly greater than 1 mm and in opposite direction of what was reported by other studies. In the present study, assessment was conducted for a mean period of 4 years and 10 months, which reduces the interference of potential changes in the maturation of occlusion on the present results. However, dimensional changes smaller than 1 mm should be carefully considered to avoid confusion with occasional changes inherent to sample aging.

The methods employed in this study, which included the use of the three-dimensional digitizer MicroScribe-3DX, a tool with proven efficacy,^15^ allowed assessment of three-dimensional changes of dental arches and possible influences caused by orthodontic treatment and surgical therapy.

Sample size was calculated based on data available in the literature,^7^ and was used to assess the hypothesis that the mean changes of a measurement between two study periods would be equal to zero. That is to say, the hypothesis that treatment performed between the two periods did not cause any average changes at a maximum significance level of 5%, minimum power of 80%, and under the condition that the mean was different from zero for at least half standard deviation. In these conditions, the minimum sample size was established at 25 patients. During the study, we decided to separate patients with Class II and Class III malocclusions in order to allow better homogenization of the sample. This resulted in two groups of 15, one of each class of patients. Power at these new conditions was calculated to confirm that they did not significantly reduce the power of the tests employed (Tables 2 and 4).

The preoperative period (T_2_-T_1_) revealed the role orthodontic treatment plays to prepare the dental arches in order to achieve normal occlusion after surgery. In general, maxillary and mandibular dental arches exhibited similar features at this period (Tables 2 and 4). Inter-premolar widths were increased at this period (from 1.69 mm to 3.29 mm) and buccal tipping, demonstrated by differences between the centroid and gingival landmarks, was very important (Tables 3 and 5). A study with similar methodology^7^ revealed that, during orthodontic preparation, W4-4 (1.5 ± 2.0) and W5-5 (1.4 ± 2.0) measured by the centroid were expanded, revealing the clear orthodontic tendency towards eliminating the natural compensation established.

The idea that mandibular inter-canine width is basically unchangeable has been repeatedly supported in the literature. Burke et al^16^ assessed stability in the mandibular inter-canine width of cases orthodontically treated with and without extractions. Their results revealed that, regardless of diagnosis and type of treatment, mandibular inter-canine width presents a tendency towards expansion in 1 to 2 mm during treatment, returning to the initial dimensions after the retention period. Our results revealed that inter-canine width remained stable for the mandibular arch at the three study periods. Conversely, the maxillary arch increased in the orthodontic period (centroid 1.72 mm and gingival 1.23 mm) with stability in the postoperative period. Similar results were described by Martin^7^ who observed an increase in the maxillary W3-3 of 0.7 ± 2.1 from the centroid landmark, during the orthodontic period. Ward et al^17^ observed that, from 20 to 31 years of age, small increases occur in maxillary and mandibular intercanine widths (+0.22 and +0.05, respectively).

In the mandibular arch, the distance between second molars measured from the centroid landmark reduced during orthodontic finalization (T_3_-T_2_). Despite such reduction, measurements obtained between the centroid and gingival landmarks (Table 3) at T_2_-T_1_ revealed greater movement of the centroid landmark, with opposite movement at T_3_-T_2_.

Martin^7^ observed that, during orthodontic preparation, W6-6 and W7-7 measured from the centroid landmark remained stable, differently from what was observed when measurement was performed from the gingival landmark, which revealed a reduction in W6-6 (-2.1 ± 3.0) and W7-7 (-1.6 ± 2.2). A possible explanation for this finding might be related to the presence of bands at T_2_ when measurements comparing the initial treatment period were obtained, thus impairing the correct identification of gingival landmarks and giving rise to smaller preoperative measurements . In the present study, 0.2 mm were decreased from T_2_ measurement on each side of the arch in order to avoid this interference.

The use of preformed archwires may be related to an increase in inter-premolar width, since patients with Class II division 1 malocclusion often present triangular-shaped dental arches. The greater increase observed in the maxillary arch may be related to the need to coordinate maxillary and mandibular archwires in transverse direction, since the dental arches of patients with Class II relationship tend to present posterior crossbite when changed to a Class I relationship at surgery. The surgeries performed did not include dentoalveolar segmentation so as to allow surgical correction of transverse discrepancies in three or four pieces. Even though this study did not include individuals treated with mechanical expansions, the coordination of archwires with the use of diagrams is very common during the preoperative period. Surprisingly, no transverse relapse was observed in the postoperative period (T_3_-T_2_). Considering that potentially unstable movements should be avoided during the preoperative orthodontic period,^18^ widening of dental arches in the transverse direction by expansion and buccal tipping may be an unadvisable procedure. However, the preoperative changes observed in the present study did not cause contraction of dental arches after removal of the orthodontic appliance. Conversely, solid transverse stability was observed both in the maxillary and mandibular arches. During T_3_-T_1_, three out of four measurements in the mandibular arch indicating arch expansion at period T_2_ remained positive and higher than what was observed at the onset of assessment at T_1_ (Table 2). In the maxillary dental arch, four out of six measurements indicating transverse expansion observed in preoperative orthodontics were still increased by the end of the assessment period (Table 4).

The clinical application of these findings is very important. Transverse expansions during preoperative orthodontic treatment allow adequacy of dental arch dimensions and prevent the need for maxillary segmentation, commonly used for that purpose. This would reduce the period of surgical intervention and inherent morbidity of the additional procedure. Moreover, expansion of dental arches favors the resolution of tooth crowding without affecting the incisors inclination.

These findings should be carefully interpreted. In the mandibular arch, except for first premolars, all measurements indicating inclination of posterior teeth at T_3_-T_1_, which compared the first and last evaluations of the present study, were non-significant, revealing that buccal tipping observed at T_2_ was not present at T_3_ (Table 3). In the maxillary dental arch, both transverse dimensions and buccal tipping of posterior teeth achieved by preoperative orthodontic treatment presented a tendency towards maintenance at the final study period (Tables 4 and 5). The length and depth of maxillary and mandibular dental arches remained unchanged in the study periods. This may be assigned to transverse expansion of dental arches, which was maintained throughout treatment. The only exception observed was a slight decrease (0.74 mm) in the length of the maxillary dental arch at the postoperative period (Table 4).

Future studies with longer follow-ups after the retention period, conducted with larger samples and with paired control groups, may contribute to confirm the present findings.

## CONCLUSIONS

Maxillary and mandibular dental arches presented transverse expansion with buccal tipping of maxillary and mandibular premolars and maxillary canines during preoperative orthodontic preparation of patients with Class II division 1 malocclusion. This expansion remained throughout the study period. With regards to inclination of posterior teeth, the maxillary arch presented greater stability than the mandibular arch. Further studies are necessary to confirm the present findings.
